# Targeting PIM kinases to oppose hypoxia-mediated therapeutic resistance

**DOI:** 10.18632/oncoscience.458

**Published:** 2018-08-22

**Authors:** Shailender S. Chauhan, Noel A. Warfel

**Affiliations:** University of Arizona Cancer Center, Department of Cellular and Molecular Medicine, University of Arizona, Levy Cancer Center, Tucson, Arizona 85724 USA

**Keywords:** PIM kinase, hypoxia, angiogenesis, Nrf2, therapeutic resistance

**Figure 1:**
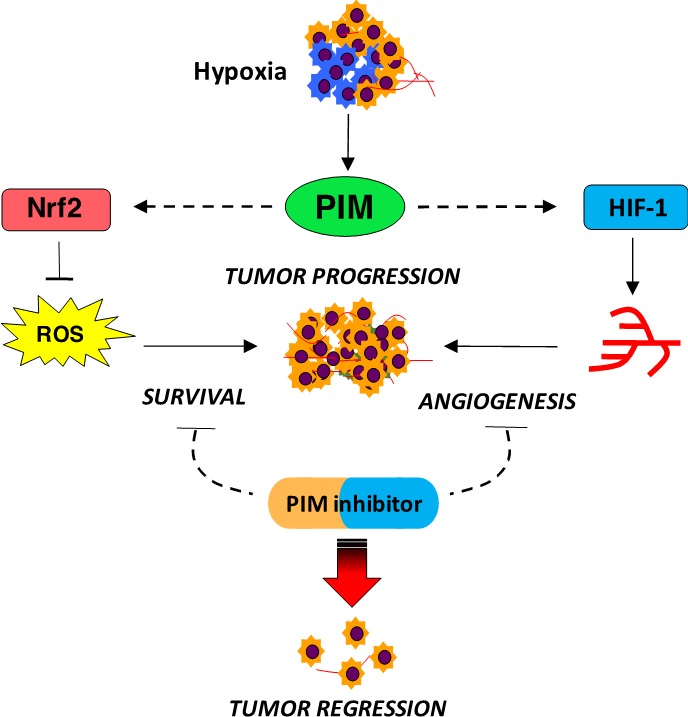
PIM kinase promotes survival and angiogenesis in response to hypoxia

PIM kinase family members have been implicated as important factors in the progression and prognosis of various malignancies, including leukemia, breast, and prostate cancer. As a result, PIM kinases are emerging as potential targe ts for solid tumors. In fact, many pharmacological inhibitors of PIM kinases are already under clinical trials [1]. A growing body of evidence suggests that PIM kinases are particularly important in the context of cellular stress, such as hypoxia. Notably, PIM kinase expression is increased in hypoxia in a HIF-1- independent manner, which makes PIM inhibition a novel approach to target the hypoxic tumor microenvironment. Recent reports highlight hypoxia-induced PIM kinase expression as a novel signal transduction pathway that provides protection against hypoxic stress by promoting survival and angiogenesis (Figure 1) [2,3].

Resistance to anti-angiogenic therapy is often attributed to the ability of hypoxic cells to maintain a proliferative phenotype [4] and initiate angiogenic compensation [5]. Casillas et al. tested the hypothesis that PIM kinase could be responsible for imparting *de novo *and/or acquired resistance to anti-angiogenic agents, as these drugs are designed to disrupt vasculature, which increases hypoxic stress [3]. Treatment with anti-VEGF targeting agents dramatically increases PIM kinase expression, and overexpression of PIM1 effectively blocks the ability of these drugs to prune tumor vasculature. Moreover, PIM inhibitors dramatically reduce tumor vasculature when combined with anti-VEGF therapies, suggesting that PIM is driving angiogenesis through a novel VEGF-independent mechanism [3]. While PIM1 is not a HIF-1 target, it appears to be an important signal for controlling the magnitude of HIF-1 signaling. PIM inhibitors significantly reduce HIF-1 activation via promoting the hydroxylation-dependent degradation of HIF-1. Thus, overexpression of PIM1, which is frequently observed in many solid tumors, regardless of hypoxia, could inhibit the canonical HIF-1 degradation pathway, representing a novel mechanism to explain the constitutive activation of HIF-1 observed in cancer.

In addition to synergistic inhibition of tumor vasculature, combined inhibition of PIM and VEGF results in enhanced cell death and a dramatic reduction in tumor cell proliferation. The expression and activation of PIM kinase during hypoxia increases the level of cytoprotective genes via Nrf2, which provides protection against reactive oxygen species (ROS)-mediated cell death [2]. Studies conducted human subjects and animal models have linked oxidative stress, ROS, and Nrf2 activation to numerous biological functions, including survival and proliferation. Nrf2 provides protection from insults such as xenobiotics and oxidative stress through activation of the cellular antioxidant response by enhancing the expression of multiple genes combating free radical-associated damage [6]. As a result, PIM indirectly regulates Nrf2 functions that promote survival in hypoxia, such as cellular redox homeostasis, NADPH generation, autophagy, apoptosis, and metabolism (heme, lipid, and glucose). Thus, targeting PIM kinases in cancer represents a suitable approach to regulate Nrf2 activation, which might create better avenues for combinatorial therapy to counteract drug resistance. Furthermore, PIM expression has been implicated in the metastatic spread of prostate cancer [7]. PIM inhibitors alone significantly reduce metastasis in orthotopic models of prostate and colon cancer [3], indicative of a potential role for PIM in promoting the invasive phenotype associated with hypoxia. Taken together, these findings demonstrate the breadth of cellular processes that PIM kinases impact and provide further evidence of their importance as drivers of therapeutic resistance in response to hypoxia.

In recent years, appreciation for the importance of the tumor microenvironment in driving resistance to standard and targeted cancer therapies has grown considerably. It is clear that in order to successfully treat solid tumors, we must target both genetic and environmental factors that allow tumor cells to evade therapy and progress to metastatic disease. PIM kinases are emerging as a critical, selective, and druggable target to oppose hypoxia-mediated therapeutic resistance in cancer. Considering that PIM inhibitors are actively being pursued in clinical trials, surprisingly little is known about how the expression and activity of these kinases are regulated. Thus, further efforts are warranted to understand how PIM kinases influence (and are influenced by) the tumor microenvironment, as well as their multifaceted role in promoting the aggressive, resistant phenotype associated with tumor hypoxia. A better understanding of how PIM kinase expression and activity are controlled in hypoxia to propagate hypoxic signaling will undoubtedly provide new and effective contexts in which PIM inhibitors might overcome therapeutic resistance in solid tumors.
